# Is there life on the airway tree? A pilot study of bronchial cell vitality and tissue morphology in the ex vivo lung perfusion (EVLP) era of lung transplantation

**DOI:** 10.1111/aor.14342

**Published:** 2022-06-28

**Authors:** Massimo Boffini, Paola Cassoni, Alessandro Gambella, Erika Simonato, Luisa Delsedime, Matteo Marro, Vito Fanelli, Andrea Costamagna, Paolo Olivo Lausi, Paolo Solidoro, Fabrizio Scalini, Cristina Barbero, Luca Brazzi, Mauro Rinaldi, Luca Bertero

**Affiliations:** ^1^ Cardiac Surgery Division, Department of Surgical Sciences University of Turin Turin Italy; ^2^ Pathology Unit, Department of Medical Sciences University of Turin Turin Italy; ^3^ Pathology Unit, AOU Città della Salute e della Scienza University Hospital Turin Italy; ^4^ Department of Anesthesia and Intensive Care Medicine, Department of Surgical Sciences University of Turin Turin Italy; ^5^ Thoracic Surgery Division, Department of Surgical Sciences University of Turin Turin Italy; ^6^ Pneumology Division, Department of Medical Sciences University of Turin Turin Italy

**Keywords:** airway tree, cell vitality, ex vivo lung perfusion, ischemic‐reperfusion injury, lung transplant, regenerative medicine

## Abstract

**Background:**

Ex vivo lung perfusion (EVLP) is a relevant procedure to increase the lung donor pool but could potentially increase the airway tree ischemic injury risk.

**Methods:**

This study aimed to evaluate the direct effect of EVLP on the airway tree by evaluating bronchial cell vitality and tissue signs of injury on a series of 117 bronchial rings collected from 40 conventional and 19 EVLP‐treated lung grafts. Bronchial rings and related scraped bronchial epithelial cells were collected before the EVLP procedure and surgical anastomosis.

**Results:**

The preimplantation interval was significantly increased in the EVLP graft group (*p* < 0.01). Conventional grafts presented cell viability percentages of 47.07 ± 23.41 and 49.65 ± 21.25 in the first and second grafts which did not differ significantly from the EVLP group (first graft 50.54 ± 25.83 and second graft 50.22 ± 20.90 cell viability percentage). No significant differences in terms of histopathological features (edema, inflammatory infiltrate, and mucosa ulceration) were observed comparing conventional and EVLP samples. A comparison of bronchial cell viability and histopathology of EVLP samples retrieved at different time intervals revealed no significant differences. Accordingly, major bronchial complications after lung transplant were not observed in both groups.

**Conclusions:**

Based on these data, we observed that EVLP did not significantly impact bronchial cell vitality and airway tissue preservation nor interfere with bronchial anastomosis healing, further supporting it as a safe and useful procedure.

## Graphical abstract

Graphical representation of the study design, procedures, and results. Cases were retrieved to represent conventional and EVLP grafts.

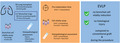

## 1 INTRODUCTION

Lung transplantation (LTx) represents the best therapeutic approach to several end‐stage lung diseases, such as cystic fibrosis, advanced chronic obstructive pulmonary disease, idiopathic interstitial pneumonia and interstitial lung disease, alpha‐1 antitrypsin deficiency‐related emphysema, and idiopathic pulmonary arterial hypertension.^1^, ^2^ Similar to other solid organ transplants, LTx suffers from a limited donor pool and it is burdened by early phase and long‐term complications, such as ischemic‐reperfusion injury (IRI), primary graft dysfunction (PGD), infection, rejection, and chronic lung allograft dysfunction.^1^, ^2^, ^3^, ^4^ In particular, IRI is a harmful condition harboring the potential to induce PGD and increase patient overall morbidity, mortality, and long‐term complications such as chronic lung allograft dysfunction.^5^ IRI is defined as aseptic inflammatory damage of the graft due to mitochondrial injury and reactive oxygen species release.^6^ It begins as a hypoxic status characterized by ischemic injury due to the abrupt and prolonged perfusion interruption. Once the organ is implanted and the vascular structures anastomosed, the blood reperfusion increases the severity of cell and tissue injury since oxygen restoration induces the activation of inflammatory cells and the production of reactive oxygen species that further injure the ischemic organ.^5^, ^7^ IRI has been widely evaluated in different types of graft transplants and encouraging results in preventing its development are increasingly reported, particularly thanks to the introduction of ex vivo machine perfusion technology.^5^, ^7^, ^8^, ^9^

In the LTx context, Ex Vivo Lung Perfusion (EVLP) emerged as a revolutionary technique to preserve, recondition, and eventually treat lung grafts before implantation, ultimately increasing the pool of donors.^10^, ^11^, ^12^ As different EVLP procedures have been proposed, the Toronto protocol proved to be particularly efficient and widely adopted. It is based on acellular solution perfusion (Steen solution) keeping the left atrium closed after a period of cold ischemic storage.^13^ Overall, EVLP allowed recovering of suboptimal donor grafts that, after reconditioning, presented similar outcomes compared with conventional grafts, as observed in several published clinical trials evaluating EVLP performances.^10^, ^14^, ^15^ Of note, during EVLP, the lung parenchyma still suffers from ischemic injury, but it is ventilated and perfused in an ex vivo controlled environment, thus partially offsetting the IRI development and reducing its impact after graft implantation in the recipient.^16^, ^17^ However, during the procedures, the airway tree is not perfused and thus fully exposed to IRI effects. Additionally, compared with standard graft procedures, the airway tree of EVLP‐treated lungs experiences a longer period of ischemic time due to the overall increased procedure duration. This potential damage is particularly crucial since airway anastomotic complications can significantly hamper the performance and overall yield of LTx.^16^, ^17^

In this setting, our study aimed to assess and grade the effect of prolonged ischemia and IRI on the airway tree of EVLP‐treated lung grafts and compare these findings with the outcomes observed in conventional grafts.

## 2 METHODS

This is a retrospective study based on 59 consecutive bilateral lung grafts collected at the AOU Città della Salute e della Scienza Hospital, Turin, Italy, from June 2015 to January 2018. Cases were selected to represent both conventional (*n* = 40) and EVLP‐treated (*n* = 19) grafts. Conventional grafts were managed according to our Transplant Surgical Unit routine procedure, whereas EVLP was performed according to the Toronto protocol (cold storage, acellular normothermic perfusion with Steen Solution, and closed left atrium), as previously reported by our group and further detailed below.^18^, ^19^, ^20^

Airway tissue rings were collected from all the 59 grafts as follows:

- Conventional graft: bilateral bronchial rings were collected before the surgical anastomosis (1 specimen/bronchus per graft);
- EVLP graft: bilateral bronchial rings were collected before ex vivo perfusion and before the surgical anastomosis during the transplant procedure;

Overall, 117 bronchial rings were collected (1 bronchial ring was lost during the retrieval). Once completed the tissue ring retrieval, airway anastomoses were performed according to our Transplant Surgical Unit routine procedure, thus using a running suture with a 3/0 mid‐term absorbable synthetic monofilament suture. Then, the suture was covered with nearby tissue without direct revascularization.

**FIGURE 1:**
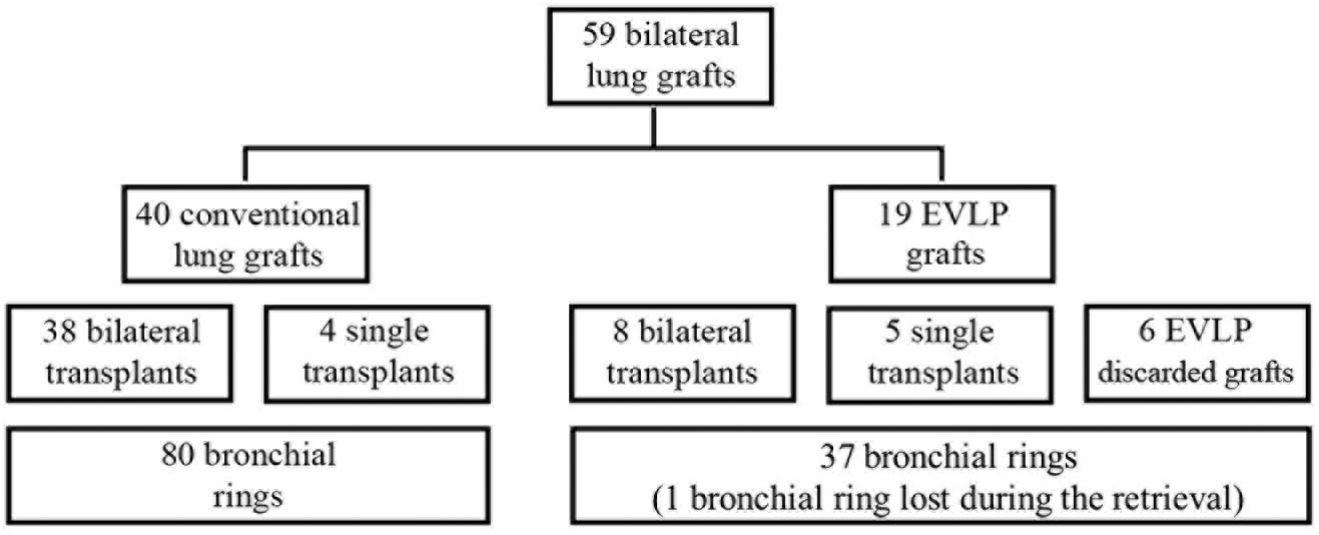
Graft stratification according to the procedure employed (conventional vs. EVLP) and their final utilization.

EVLP grafts that did not meet the criteria for transplant after the EVLP procedure (*n* = 6) were discarded. Still, we sampled these cases as follows: bilateral bronchial rings were collected before ex vivo perfusion as per the utilized grafts, whereas the second bronchial ring sample (the one that was obtained at the time of the airway anastomosis in the transplanted grafts) was collected after 1 h at room temperature (as per single transplant) or after 2 h of cold storage and 1 h of room air temperature (as per bilateral transplant) to mimic the transplant procedure timing. Conventional and EVLP grafts stratification and final utilization are summarized in Figure 1.

### 2.1 EVLP procedure

Initially rejected grafts with poor gas exchange due to pulmonary edema, but without evidence of infection, *ab ingestis* pneumonia, contusions, or structural parenchymal alterations have been retrieved and preserved according to standard protocols, thus using antegrade and retrograde flush of Perfadex and cold storage at 4°C. Grafts have been transferred to our center to be perfused and ventilated according to the Toronto Lung Transplant Group protocol.^13^ The trachea has been intubated for ventilation, pulmonary artery, and left atrium cannulated and connected to a perfusion circuit primed with Steen Solution. Body temperature and full flow of perfusion (40% of the ideal cardiac output) have been reached in 1 h. Components of the circuit were: a set of tubes, a reservoir, an oxygenator (connected to a tank of a gas mix of 86% N2, 8% CO_2_, and 6% O_2_), a centrifugal pump, and an antileukocyte filter. Protective ventilation (tidal volume 7 ml/kg, respiratory rate 7 acts/min, positive‐end expiratory pressure of 5 cm H_2_O, fraction of inspired oxygen 21%) has been started when the graft reached the temperature of 32°C. At every hour of perfusion, the ventilation setting has been modified for 5 min as follows: tidal volume 10 ml/kg, respiratory rate 10 acts/min, positive‐end expiratory pressure of 5 cm H_2_O, and fraction of inspired oxygen 100%. Bronchoscopy and lung X‐ray have been performed after 1 and 3 h of perfusion. The evaluation was based on gas exchange (pO_2_ in pulmonary veins, pO_2_ in the pulmonary vein—pO_2_ in pulmonary artery), lung dynamics (airway pressure, dynamic, and static compliance), hemodynamics (pulmonary artery and left atrium pressure), lung X‐ray, and bronchoscopy findings.

A positive reconditioning was defined according to the following parameters: delta pO_2_ (left atrium pO_2_‐pulmonary artery pO_2_): ≥350 mm Hg, left atrial pressure: from 3 to 5 mm Hg, pulmonary artery pressure: stable and <15 mm Hg, airway pressure: stable or decreased, pulmonary vascular resistance: stable or decreased, lung compliance: stable or decreased, bronchoscopy: negative, and lung X‐ray: negative. Once the graft met the criteria for transplant, it was cooled down to 10°C for 10 min and preserved in Perfadex at 4°C before implantation.

### 2.2 Airway cell vitality assay

Bronchial ring cell vitality was evaluated according to our pathology laboratory validated protocol, as previously published.^21^

Briefly, airway tissue samples were stored in sterile Falcon tubes (F1) prefilled with Roswell Park Memorial Institute (RPMI) medium supplemented with Penicillin–Streptomycin‐Fungizone (PSF) at 4°C immediately after their retrieval. Then, tissue rings were washed in a sterile Petri dish (100 mm) with 5 ml of RPMI + PSF s.f. medium and eventually scraped with a blade to collect viable cells. An additional part of the inner mucosa was also collected. The medium containing the cells and the inner mucosa was added to F1 and then supplemented with 2.5 ml of collagenase IV and shaken to enzymatically digest mucosa fragments. The medium was incubated at 37°C for at least 1 h and regularly shaken until the solution was free of fragments. Then the F1 were stored under a sterile hood, and a 10 ml of complete “Dulbecco's Modified Eagle Medium: Nutrient Mixture F‐12” (DMEM + F12) culture medium was added to block the action of collagenase. After centrifugation at 800 RPM for 5 min, the supernatant was removed by aspiration, and the cell pellet was resuspended in a volume of complete DMEM + F12 medium depending on the quantity of the obtained material (2 ml). To perform the cell count, an aliquot of cell suspension (50 μl) was collected and transferred into an Eppendorf tube. A 1:1 dilution with 50 μl of cell suspension and 50 μl of Trypan Blue dye was prepared, thoroughly resuspending the sample with a micropipette.

About 20 μl of the sample were loaded into a hemocytometer (Bürker chamber), where the sample spreads by capillary action. By using a phase‐contrast microscope, we proceeded with the cell count. The hemocytometer provides a standardized grid made of 9 squares; the average number of cells that are counted in the 9 squares is multiplied by a fixed value that is 104 (since each square represents a total volume of 0.1 mm^3^) to obtain the number of cells (live and dead) in 1 ml of solution.

**FIGURE 2:**
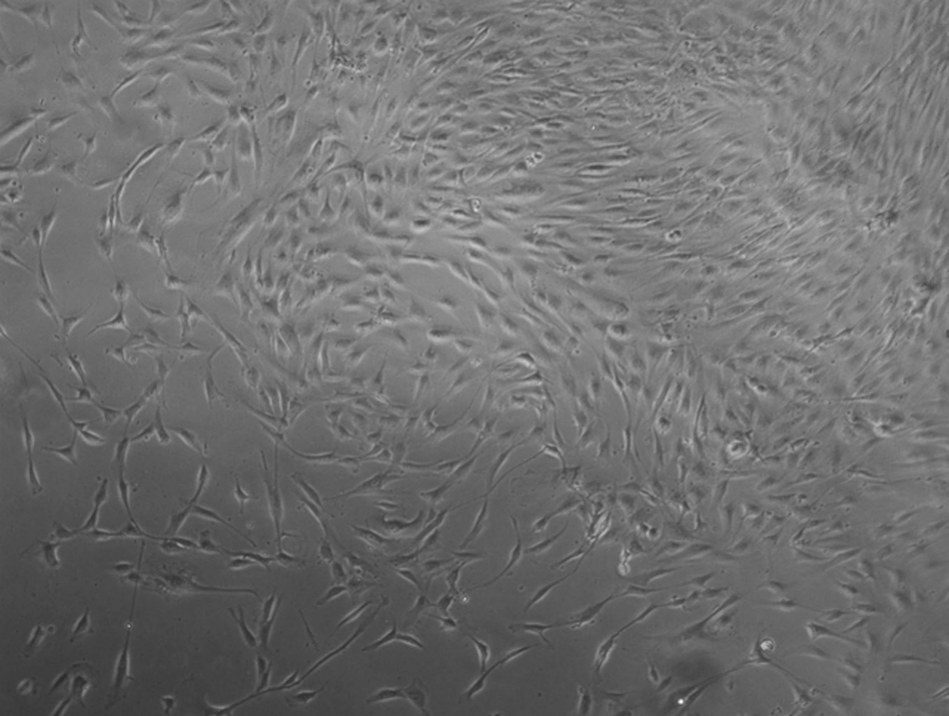
EVOS inverted microscope images (400× original magnification). Primary cell cultures, adhering to the plate, obtained from the inner mucosa of bronchial rings after EVLP procedures are shown.

The remaining cell suspension was dispensed in a 6‐well plate, checking cell density under a phase‐contrast microscope, and the multi‐well was placed in an incubator at 37°C with 5% of CO_2_. In the following days, the cell culture was monitored to check that no bacterial or fungal contamination occurred. Photographs were taken with an EVOS fl microscope (Figure 2). Finally, the cells have been frozen when they arrived at the confluence.

### 2.3 Bronchial histological assessment

**FIGURE 3:**
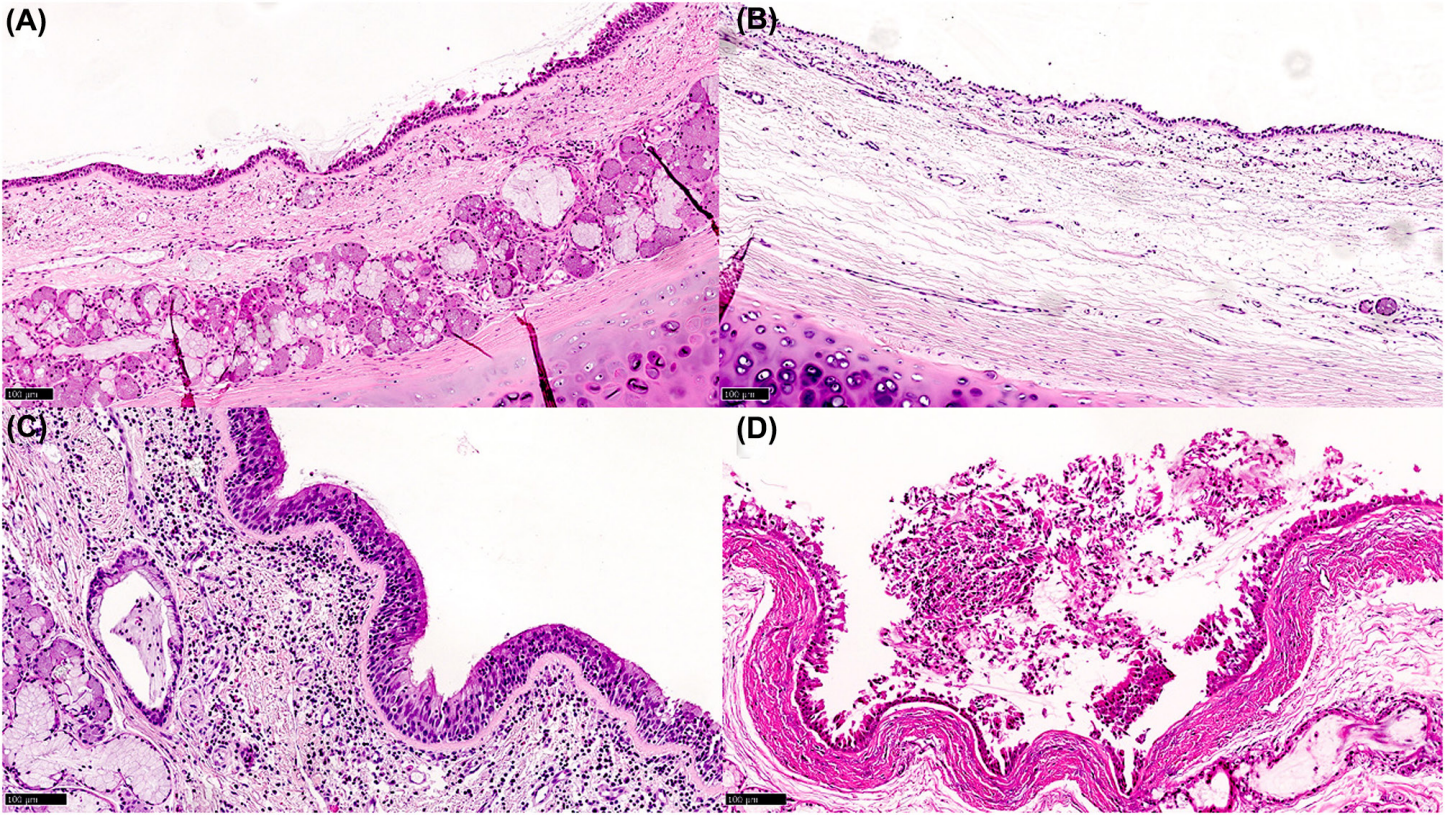
Histological samples of bronchial mucosa. Representative images of the evaluated histological features in the conventional (A) and EVLP (B–D) groups (hematoxylin and eosin, 400× original magnification). (A) normal mucosa, (B) focal and prominent edema (grade 2), (C) diffuse and moderate inflammatory infiltrate (grade 2), (D) focal mucosal ulceration (grade 1). [Color figure can be viewed at wileyonlinelibrary.com]

After scraping and mucosal sampling, airway tissue rings were transferred to a new sterile 50 ml falcon tube with 10% neutral buffered formalin for 24‐h tissue fixation. Samples were then paraffin‐embedded, processed, and 5‐μm sectioned according to the Pathology Unit laboratory routine protocols. Tissue slides were stained with hematoxylin and eosin and assessed by three expert pathologists (A.G., L.B., and L.D.) in lung and transplant pathology. The histological injury was evaluated through the assessment of three morphological features of damage, namely edema, inflammatory infiltrate, and mucosal ulceration, grading their severity in a four‐tiered system (Table 1) and also calculating each sample's total score. Representative images of the histopathological features are illustrated in Figure 3.

**TABLE 1:** Definitions of the grading system adopted for histopathological evaluation

Pathology feature	Grade 0	Grade 1	Grade 2	Grade 3
Edema	Absent	Focal and mild/moderate	Focal and prominent or diffuse and mild/moderate	Diffuse and prominent
Inflammatory infiltrate	Absent	Focal and mild/moderate	Focal and prominent or diffuse and mild/moderate	Diffuse and prominent
Mucosal ulceration	Absent	Involved <25% of the bronchial wall	Involved ≥25 and <50% of the bronchial wall	Involved ≥50% of the bronchial wall

### 2.4 Statistical analysis

Continuous variables were reported as means ± standard deviations (SD) or medians with ranges, whereas categorical variables were reported as counts. Comparisons between groups were performed using a *χ* ^2^ test or ANOVA test as appropriate. Results were considered statistically significant for the *p* value of <0.05. Statistical analyses were performed with the Stata 15.0 statistical software (StataCorp, College Station, TX, USA).

## 3 RESULTS

### 3.1 Characteristics of the series

Regarding donors’ clinical characteristics and graft procedures, the only variables that significantly differed between samples that underwent the conventional protocol and EVLP procedure were the gas exchange in terms of PaO_2_/FiO_2_ at 100% oxygen (*p* < 0.05) and the out‐of‐the‐body interval due to the additional time required by the EVLP procedure (*p* < 0.01). Conversely, we observed no significant differences in terms of ischemic time between the two groups. Of note, the EVLP procedure mean time (±SD) was 273.6 ± 64.80 min. The clinical characteristics of our donor pool are summarized in Table 2.

**TABLE 2:** Characteristics of donors and graft procedures stratified according to transplant protocol

			Conventional grafts (*n* = 40)	EVLP grafts (*n* = 19)	*p* value
Age	Years (mean ± SD)		41.79 ± 15.80	40.15 ± 12.87	0.7
Gender	Female		23	11	0.9
	Male		17	8	
Smoking history	Yes		8	8	0.08
	No		32	11	
Mechanical ventilation^a^	Days (mean ± SD)		5.43 ± 4	3.75 ± 2.59	0.09
Traumatic death	Yes		10	2	0.2
	No		30	17	
Gas exchange^b^	PaO_2_/FiO_2_ at 100% oxygen (mean ± SD)		478.86 ± 107.11	299.6 ± 122.02	<0.05
Out‐of‐the‐body time^c^	Minutes (mean ± SD)	First graft	261.71 ± 60.40	667.15 ± 150.70	<0.01
		Second graft	370.30 ± 60.20	781.4 3 ± 183.40	

^a^ Mechanical ventilation refers to the time donors were ventilated before organ procurement.

^b^ The gas exchange (PaO_2_/FiO_2_ ratio) of the EVLP and conventional group refers to the value recorded during the evaluation of organ procurement suitability.

^c^ Corresponds to the ischemic time for the conventional grafts, whereas it represents both the ischemic time and the EVLP procedure duration for the EVLP grafts.

All conventional lung grafts were transplanted, 38 as bilateral and 4 as single transplants. Bilateral bronchial rings were retrieved from each sample (Figure 1). Differently, 6 of the EVLP grafts did not meet the criteria for transplant and were discarded. Still, we sampled these cases for airway tree tissue rings as reported in the Methods section (Figure 1).

In the posttransplant follow‐up period, no anastomosis dehiscence events have been recorded in both groups, whereas we observed two cases of mild stenosis in conventional grafts follow‐up, which occurred 6 and 8 months after transplant, respectively, and did not require any specific treatment. Additionally, no evident modifications or new onset of injury were noticed in mid‐ and long‐term follow‐up bronchoscopy (median follow‐up: 36 months) in both groups. However, no statistically significant differences were observed between the two groups. Clinical characteristics of recipients and postoperative major events are reported in Table 3.

**TABLE 3:** Clinical characteristics of recipients and postoperative major events stratified according to the transplant procedure

		Conventional grafts (*n* = 42)	EVLP grafts (*n* = 13)^a^	*p* value
Age	Years (mean ± SD)	51.59 ± 13.43	50 ± 15.35	0.7
BMI (mean ± SD)		23.91 ± 4.79	25 ± 4.26	0.5
Gender	Female	16	5	0.9
	Male	26	8	
Pulmonary fibrosis	Present	16	7	0.2
	Absent	26	6	
Cystic fibrosis	Present	8	1	0.3
	Absent	34	12	
COPD	Present	13	1	0.09
	Absent	29	12	
Preoperative ECMO	No	42	12	0.07
	Yes	0	1	
LTx	Single	4	5	0.3
	Bilateral	38	8	
CPB	Yes	6	4	0.3
	No	36	9	
Postoperative ECMO	Yes	2	3	0.1
	No	40	10	
Anastomosis dehiscence	Yes	0	0	–
	No	42	13	
Mild airway stenosis	Yes	2	0	0.6
	No	40	13	

Abbreviations: COPD, chronic obstructive pulmonary disease; CPB, cardiopulmonary by‐pass; ECMO, extracorporeal membrane oxygenation.

^a^ Data regarding excluded EVLP grafts are not reported in the table.

### 3.2 EVLP did not hamper airway cell vitality

We observed that bronchial cell vitality did not differ significantly between conventional and EVLP grafts, both considering the first and second graft (*p* = 0.58 and *p* = 0.85, respectively). Additionally, no significant differences were observed within each group, as the first and second grafts of the conventional group presented a mean ± SD percentage of bronchial cell vitality of 47.07 ± 23.41 and 49.65 ± 21.25 (*p* = 0.61). Similarly, in the EVLP graft group, bronchial cell vitality was similar before EVLP and between the first and the second implanted graft (*p* = 0.34, *p* = 0.38, and *p* = 0.97, respectively).

The outcomes of the bronchial cell vitality analysis are summarized in Tables 4 and 5.

**TABLE 4:** Bronchial vital cells distribution among grafts

	Conventional grafts	EVLP grafts	*p* value
Pre‐EVLP	–	57.18 ± 27.71	–
First graft	47.07 ± 23.41	50.54 ± 25.83	0.58
Second graft	49.65 ± 21.25	50.22 ± 20.90	0.85

*Note*: Values referred to the percentage of vital cells ± SD.

Abbreviation: EVLP, ex vivo lung perfusion.

**TABLE 5:** Bronchial vital cells distribution within graft group

	Pre‐EVLP (A)	First graft (B)	Second graft (C)	*p* value
Conventional grafts	–	47.07 ± 23.41	49.65 ± 21.25	0.61
EVLP grafts	57.18 ± 27.71	50.54 ± 25.83	50.22 ± 20.90	0.38 (A vs. B); 0.34 (B vs. C); 0.97 (A vs. C)

*Note*: Values referred to the percentage of vital cells ± SD.

Abbreviation: EVLP, ex vivo lung perfusion.

### 3.3 EVLP is not associated with histological damage

No significant differences were observed when comparing each single feature (edema: *p* = 0.92, inflammatory infiltrate: *p* = 0.58, mucosal ulceration: *p* = 0.43) or the total score (*p* = 0.87) between the conventional and the EVLP graft samples (Figure 2). Similarly, comparison within the EVLP group showed no significant differences between pre‐EVLP and post‐EVLP (both first and second graft) samples (edema: *p* = 0.10, inflammatory infiltrate: *p* = 0.63, mucosal ulceration: *p* > 0.99, and total score: *p* = 0.38). Results of the histological evaluation are reported in Table 6.

**TABLE 6:** Pathology evaluation of airway samples

	Conventional grafts	EVLP grafts	*p* value	Pre‐EVLP	EVLP first graft	EVLP second graft	*p* value
Edema	1 (0–3)	1 (0–2)	0.92	1 (0–2)	1 (0–2)	1 (1–2)	0.10
Inflammatory infiltrate	1 (0–3)	1 (0–3)	0.58	1 (0–2)	1 (0–3)	1 (0–3)	0.63
Mucosal ulceration	0 (0–1)	0 (0–2)	0.43	0 (0–1)	0 (0–1)	0 (0–2)	0.98
Total score	2 (1–6)	2 (0–6)	0.87	2 (0–4)	2 (0–5)	2.5 (1–6)	0.38

## 4 DISCUSSION

Our study found that EVLP did not reduce the airway cell vitality, nor it induces evident morphological signs of injury of the corresponding mucosa, regardless of the increased ischemic time experienced by the airway tree during the EVLP procedure compared with a conventional graft. This evidence further supports the safety of EVLP and represents a significant baseline for further granular analysis of EVLP effect on graft airway.

Indeed, EVLP is a relatively recent procedure that has proved to (1) increase lung parenchyma recruitment, (2) reduce lung edema incidence and severity and improve graft gas exchange performance, thanks to the hyperoncotic solution perfusion, and (3) allow to implement specific treatments (e.g., antibiotics), ultimately increasing the donor graft pool.^10^, ^15^, ^18^, ^19^, ^20^, ^22^, ^23^, ^24^, ^25^, ^26^, ^27^ Clinical trial results further supported its adoption by showing equal outcomes of EVLP‐treated grafts compared with the conventional approach.^14^, ^28^, ^29^, ^30^, ^31^, ^32^, ^33^ However, some aspects of EVLP are still debated, such as the best protocol to be used, the limits and consequences of graft manipulation, and the effect on bronchial ischemia and anastomotic complications.

To date, a variable degree of ischemic injury (i.e., bronchial necrosis, dehiscence, or most frequently stenosis) in the donor bronchial stump frequently occurred^34^, ^35^ and bronchial anastomotic complications still represent a significant cause of LTx morbidity and mortality, occurring in 2%–32% of cases.^33^, ^34^, ^36^, ^37^ In this setting, we reached out to assess the hypothesis that the airway of lung grafts may be exposed to increased ischemic injury during EVLP.

Indeed, the EVLP‐treated graft airway experiences prolonged vascularization interruption compared with a conventional graft, thus some concerns may arise regarding bronchial vitality and bronchial anastomosis healing. The Toronto Lung Transplant Group reported an equal incidence of bronchial healing complications between EVLP‐treated grafts and conventional grafts (4% in each group),^14^ suggesting that EVLP does not hamper the bronchial healing process. In our study, we provided evidence supporting this hypothesis. To the best of our knowledge, our study is the first to investigate bronchial vitality and morphological features of graft airway during the EVLP procedure.

In our study, we did not report any impact of EVLP compared with conventional grafts on bronchial status, both in terms of cell vitality and histopathological signs of tissue injury. Additionally, we observed no differences in terms of injury and cell vitality at different EVLP procedure timepoints, suggesting that the time spent in EVLP according to the Toronto Protocol is safe enough for tissue preservation. This is relevant since the optimal duration of EVLP is still not ascertained, striving to identify the minimal effective time and the maximal safe duration. This way, we confirmed the Toronto Protocol timeframe’s safety and utility and defined baseline values regarding cell and tissue vitality that could serve for further studies assessing prolonged EVLP duration. This evidence is particularly significant, as increasing research protocols are exploring the opportunity to provide therapeutical support to the graft during EVLP (e.g., gene and antibiotic therapy), but these approaches require prolonged procedure extent.^22^, ^25^, ^26^, ^27^ Indeed, in other transplant settings, ex vivo machine perfusion has already proved to represent a reliable technique to provide specific treatments aiming to improve graft functionality.^38^, ^39^, ^40^ In the EVLP setting, this approach still needs to be thoroughly explored, but we believe that our data could represent a significant starting point.^22^, ^25^, ^26^, ^27^

Our study presents a few limitations: the digestion and cell isolation process could have affected cell viability, although it would have equally affected both types of graft. Moreover, this event was not observed in our previous study using the same protocol, although performed in a different context.^21^ Additionally, it would be interesting to explore signs of cell sufferance with additional methods (such as metabolic pathway activation/impairment and ultrastructural signs of mitochondrial injury) or evaluate airways functionality through additional assays (e.g., assessment of mucus production and characteristics, ciliary beating), both in the donors and recipients, and focusing on challenging clinical settings (extended EVLP protocols or high‐risk donors). This way, a more granular analysis of cell and tissue functionality during the EVLP procedure will be provided.

In conclusion, our exploratory study provides tissue‐tethered evidence that EVLP did not impact airway vitality and functionality, supporting its clinical implementation and providing a relevant baseline for further experimental investigation in the setting of lung transplantation.

## AUTHORS’ CONTRIBUTION

Concept/design: Massimo Boffini, and Paola Cassoni. Data analysis/interpretation: Massimo Boffini, Paola Cassoni, Alessandro Gambella, Erika Simonato, Luisa Delsedime, Matteo Marro, Vito Fanelli, Andrea Costamagna, Paolo Olivo Lausi, Paolo Solidoro, Fabrizio Scalini, Cristina Barbero, Luca Brazzi, Mauro Rinaldi, and Luca Bertero. Drafting the article: Massimo Boffini, Paola Cassoni, Alessandro Gambella, Mauro Rinaldi, and Luca Bertero. Critical revision of the article: Massimo Boffini, Paola Cassoni, Mauro Rinaldi, and Luca Bertero. Approval of the article: Massimo Boffini, Paola Cassoni, Alessandro Gambella, Erika Simonato, Luisa Delsedime, Matteo Marro, Vito Fanelli, Andrea Costamagna, Paolo Olivo Lausi, Paolo Solidoro, Fabrizio Scalini, Cristina Barbero, Luca Brazzi, Mauro Rinaldi, and Luca Bertero. Data collection: Massimo Boffini, Paola Cassoni, Alessandro Gambella, Erika Simonato, Luisa Delsedime, Matteo Marro, Vito Fanelli, Andrea Costamagna, Paolo Olivo Lausi, Paolo Solidoro, Fabrizio Scalini, Cristina Barbero, Luca Brazzi, Mauro Rinaldi, and Luca Bertero.

## CONFLICT OF INTEREST

The authors declare that the research was conducted in the absence of any commercial or financial relationships that could be construed as a potential conflict of interest.

## STATEMENT OF HUMAN AND ANIMAL RIGHTS

All procedures were in accordance with the ethical standards of the responsible committee on human experimentation (national and institutional) and with the World Medical Association Declaration of Helsinki of 1964 and later versions. The study was approved by the Institutional Ethical Committee (CEI‐178 and DSM‐ChBU 4/2022).

## Data Availability

The data presented in this study are available on request from the corresponding author. The data are not publicly available due to privacy restrictions.
